# Analysis of the effect of phototherapy on intestinal probiotics and metabolism in newborns with jaundice

**DOI:** 10.3389/fped.2022.878473

**Published:** 2022-10-06

**Authors:** Sainan Fan, Kun Zhang, Jiahui Zhang, Lei Zhang, Lixiao Liu, Anping Lv, Yanan Ma, Xiaohui Fang, Fang Zheng, Zhimin Wu, Jinping Zhang

**Affiliations:** ^1^1Department of Pediatrics, Shanghai Sixth People’s Hospital, Shanghai Jiao Tong University School of Medicine, Shanghai, China; ^2^Department of Pediatrics, Shanghai Pudong New Area People’s Hospital, Shanghai, China; ^3^Department of Pediatrics, Shanghai Pudong Hospital, Shanghai, China

**Keywords:** jaundice, phototherapy, probiotics, bile acids, short chain fatty acids, metabolites

## Abstract

**Background:**

In clinical practice, oral probiotics are often given to children with hyperbilirubinaemia who receive phototherapy, but the exact mechanism of the action of the probiotics on hyperbilirubinaemia remains unclear. It is unclear how the effects of phototherapy on the probiotic flora in the neonatal gut, in particular.

**Materials and methods:**

Fifty newborns who needed phototherapy from June 2018 to June 2020 were selected as the study subjects, and five healthy newborns in the same period were used as controls to analyse the changes in probiotic bacteria in their faeces.

**Results:**

1. In the intestinal tracts of newborns, *Bifidobacterium* is the main probiotic strain, with a small amount of *Lactobacillus*. There were probiotic species changes in the neonatal intestinal microbiota after phototherapy for 24 and 48 h. The amount of *Bifidobacterium* and *Lactobacillus* decreased significantly (*P* < 0.05). 2. A correlation analysis of probiotic species and bile acid metabolism indexes showed that *Bifidobacterium* was positively correlated with many metabolites (*P* < 0.05), such as chenodeoxycholic acid, hyodeoxycholic acid, cholic acid, allocholic acid, and β-cholic acid. It was also negatively correlated with many metabolites (*P* < 0.05), such as glycocholic acid, sodium, sodium tudca, and chenodeoxycholic acid. *Lactobacillus* was negatively correlated with metabolites (*P* < 0.05) such as α-sodium cholate and β-cholic acid. 3. A correlation analysis between the changes in probiotics and intestinal short-chain fatty acid metabolites after phototherapy showed that acetic acid, butyric acid, caproic acid, and propionic acid decreased and were significantly correlated with *Bifidobacterium* (*P* < 0.05). 4. After phototherapy, 17 metabolites changed significantly (*P* < 0.05). This correlated with many probiotics (*P* < 0.05). The significantly changed probiotics in this study showed a significant correlation with some intestinal metabolites (*P* < 0.05).

**Conclusion:**

It was found that phototherapy can significantly affect the intestinal probiotic flora and the metabolic indicators of newborns, which may be an important reason for the side effects of phototherapy, and also provides the theoretical basis for the provision of probiotics to newborns with jaundice.

## Introduction

Hyperbilirubinaemia is a common clinical problem in the neonatal period. It is caused by excessive bilirubin production *in vivo*, the insufficient ability of the liver to absorb and combine bilirubin, increased intestinal-liver circulation, and abnormal bilirubin excretion. The clinical manifestation is jaundice (1). About 60% of full-term and 80% of premature infants may have jaundice to different degrees in the early postnatal period (2). Neonatal jaundice is mainly mild to moderate, but when the level of unbound bilirubin is too high, it can cause bilirubin encephalopathy through the blood–brain barrier; if not treated, it causes permanent damage. At present, phototherapy is the most routine treatment for pathological jaundice. Phototherapy can change the structure of bilirubin and increase its excretion, but some long-term and short-term side effects are increasingly recognised by clinicians (3). In clinical practice, oral probiotics are often given to children with jaundice who receive phototherapy, which can help reduce jaundice (4, 5). However, many of these probiotics lack basic research support, and their use mostly relies on the clinician’s experience and preference. Therefore, we use metagenomic sequencing to deeply study probiotic bacteria changes in the neonatal intestinal tract after phototherapy and use metabonomics to analyse the correlation between the changes in probiotic species and metabolism so as to provide a theoretical basis for newborns with clinical jaundice to receive appropriate probiotics during phototherapy.

## Materials and methods

### Clinical subjects

Newborns needed phototherapy in the East Hospital of Shanghai Sixth People’s Hospital from June 2018 to June 2020. We chose neonates who received only artificial feeding and wrote down all of the clinical information of the newborns. Healthy newborns in the same period were selected as controls, and the changes in probiotic species in stool samples were analysed.

### Phototherapy

The newborns were placed in blue light treatment boxes (model XHZ, Ningbo David Medical Devices Co., Ltd., Ningbo, China), and their eyes, perineum, and scrotum were covered with a phototherapy eye mask (Foshan Forssman Medical Technology Co., Ltd., Foshan, China, Yueshun Xiebei No.20160015) and a phototherapy diaper (Foshan Baojusheng Medical Devices Co., Ltd., Foshan, China, GB/T33280). All children received continuous phototherapy for 24 h at a wavelength of 425–475 nm, rested for 6–8 h, and continued phototherapy for 24 h. Then, it was decided whether to continue treatment for the next 24 h based on the change in jaundice levels.

### Inclusion criteria

(1) The jaundice levels met the jaundice phototherapy standards of the phototherapy index of the American Pediatric Association; (2) age ≤ 2 weeks; (3) full-term infants 37–41 gestational weeks with birth weights 2,500 < 4,000 g; (4) antibiotics and ecological preparations were not used before the specimens were collected; (5) the mother was healthy during pregnancy, had no history of special drug use, and did not take antibiotics or microecological agents before, during, or after delivery; (6) Infants were fed artificially before admission; and (7) voluntary signing of informed consent.

### Exclusion criteria

(1) Gestational age < 37 weeks or ≥ 42 weeks; (2) the bilirubin level was as high as the exchange standard (20 mg/dl) or was directly elevated; (3) pneumonia, sepsis, or other diseases; (4) severe immunodeficiency; (5) hereditary metabolic diseases; (6) congenital biliary malformation or other organ malformation; and (7) drug allergies. (8) In some cases, enrolment was adjusted based on the judgement of the researcher. For example, if the guardian had a mental illness or his/her living or working environment often changed, follow-up may not have been possible.

### Ethics

This study was approved by the ethics committee of the East Hospital of Shanghai Sixth People’s Hospital (Approval No:2020-071). All family members signed the informed consent form.

### Specimen collection

The stool collection was divided into three time points: before phototherapy and 24 and 48 h after phototherapy. Meanwhile, the stool samples of the control newborns were collected on the fifth, sixth, and seventh days and then frozen at –80°C immediately after retention. The stool sample for each case was ≥ 500 mg.

### Metagenomic sequencing

Collecting children’s stool samples and studying the changes in intestinal probiotic flora before and after phototherapy by using macro-gene high-throughput analysis. DNA extraction and detection: The faecal DNA was extracted according to the instructions of the QIAamp Power Faecal DNA Kit (QIAGEN, Germany). Library construction and quality inspection: Qualified DNA samples were randomly broken into fragments with a length of about 350 bp by an ultrasonic breaker, and the whole library was prepared by the steps of terminal repair, adding A at the 3′ end and adding sequencing linkers, purification, fragment selection, and PCR amplification. After the library construction was completed, electrophoresis and Nanodrop were used for the preliminary quantification. Then, a Qubit quantification was carried out, and the qPCR method was used to accurately quantify the effective concentration of the library to ensure the quality of the library. Sequencing on the machine: After the inspection was qualified, different libraries were mixed according to the requirements of the effective concentration and the target off-machine data quantity, and then, Illumina HiSeq sequencing was performed. Bio-information analysis: Sequencing data were preprocessed, starting from clean reads after quality control, and SOAPdenovo was used to assemble the metagenome. Finally, the species were annotated, and the abundance was analysed.

### Metabonomics detection

To treat the stool samples, 0.3 ml of methanol was added to about 100 mg of the stool samples to precipitate protein, it was vortexed for 1 min, and it was centrifuged at 4°C for 10 min (12,000*g). The supernatant was diluted 10 times, vortexed for 1 min, and centrifuged at 4°C for 10 min (12,000*g). Bile acids were detected by LC/MS, short-chain fatty acids were detected by GC-MS, and metabolites were measured using non-targeted metabolomics.

### Statistical analysis

SPSS23 software was used for the statistical analysis of all data; the measurement data subject to normal distribution were expressed by the mean standard deviation ($\bar{x}$ ± s), and multiple groups of samples were compared with a variance analysis. A Kruskal–Wallis non-parametric rank-sum test was used for the comparison of groups that did not obey a normal distribution. The count data were expressed by frequency or percentage, and a chi-square test was used for comparison of the groups. A Spearman rank correlation analysis was used to analyse the correlation between samples. The difference was statistically significant at *P* < 0.05.

## Results

1. A total of 50 newborns participated in this research, and researchers collected the basic data of the research subjects. We selected five healthy newborns from the same time period as the controls (see Table 1).

**TABLE 1:** Basic information.

Basic information of subjects	Newborns who need phototherapy (*n* = 50)	Healthy control newborns (*n* = 5)
Gestational age (week)	38.7 ± 8.77	37.7 ± 2.77
Birth weight (kg)	3.3 ± 0.36	3.1 ± 0.46
Days of jaundice (day)	2.18 ± 1.40	–
Age (day)	7.10 ± 5.24	5.10 ± 0.24
Phototherapy time (h)	88.51	–

**FIGURE 1:**
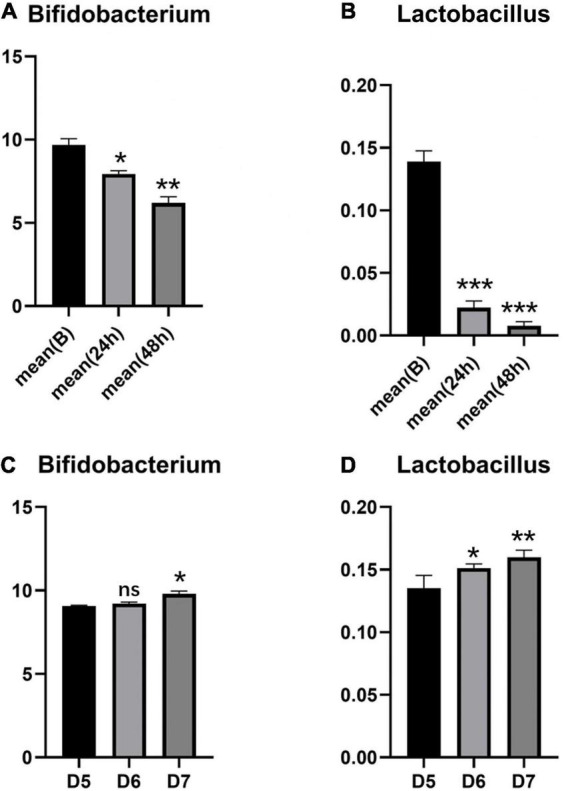
Changes of *Bifidobacterium* and *Lactobacillus* in two groups of neonates. **(A,B)** Changes of *Bifidobacteria* and *Lactobacillus* in children with phototherapy; **(C,D)** Changes of *Bifidobacterium* and *Lactobacillus* of healthy control newborns. Data are represented as means ± SD of three independent experiments. *P < 0.05, **P < 0.01, and ***P < 0.005.

**FIGURE 2:**
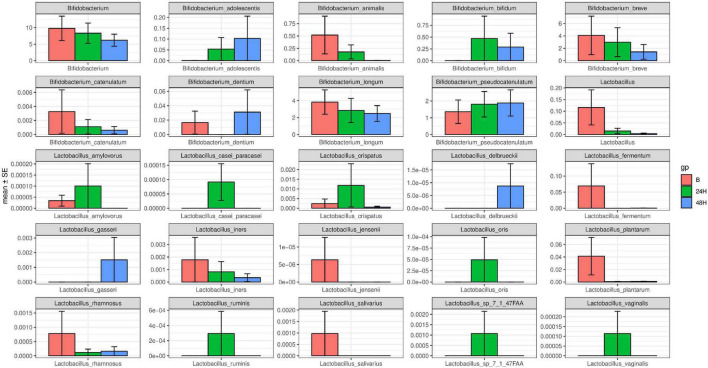
Different *Bifidobacterium* and *Lactobacillus* strains change after 24 and 48 h of phototherapy.

**FIGURE 3:**
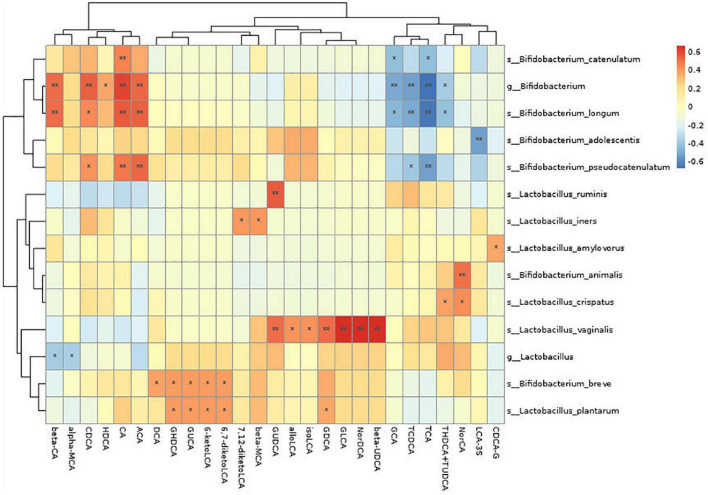
Correlation analysis between probiotic species and bile acid metabolism indexes.

2. In the intestinal tracts of newborns, *Bifidobacterium* is the main probiotic strain, and there is a small amount of *Lactobacillus*. The probiotic species changed in the neonatal intestinal microbiota after phototherapy for 24 and 48 h. *Bifidobacterium* and *Lactobacillus* decreased significantly (*P* < 0.05) (see Figures 1A,B). The specific changes of *Bifidobacterium* classifications and *Lactobacillus* classifications are shown in Figure 2. The remarkable changes occurred with *Bifidobacterium animalis*, *Bifidobacterium breve*, and *Lactobacillus fermentum*, which were significantly reduced after phototherapy (*P* < 0.05). On the 5th, 6th, and 7th days, stool samples of the five neonates were collected, and the probiotic content was measured. With the age increase, the overall content of probiotics increased (see Figures 1C,D).

3. A correlation analysis was performed for the change in probiotic species and bile acid metabolism indexes. It was found that *Bifidobacterium* was positively correlated with many metabolites (*P* < 0.05), such as chenodeoxycholic acid, hyodeoxycholic acid, cholic acid, allocholic acid, and β-cholic acid. It was also negatively correlated with many metabolites (*P* < 0.05), such as glycocholic acid, sodium taurocholate, sodium tudca, and chenodeoxycholic. *Lactobacillus* was negatively correlated with metabolites (*P* < 0.05) such as α-sodium cholate and β-cholic acid (see Figure 3 and Table 2).

**TABLE 2:** Correlation between bile acid and probiotics.

	Positive correlation bile acid	Correlation coefficient R	*P*-values	Negative correlation bile acid	Correlation coefficient R	*P*-values
*Bifidobacterium*	CDCA	0.54	0.002	GCA	–0.50	0.005
	HDCA	0.36	0.05	THDCA + TUDCA	–0.39	0.03
	CA	0.60	0.0004	TCDCA	–0.53	0.002
	ACA	0.52	0.003	TCA	–0.67	0.001
	Beta-CA	0.51	0.004			
*Lactobacillus*				Alpha-MCA	–0.37	0.05
				Beta-CA	–0.39	0.04
*Bifidobacterium breve*	6-ketoLCA	0.39	0.03			
	DCA	0.39	0.04			
	6,7-diketoLCA	0.39	0.03			
	GUCA	0.39	0.03			
	GHDCA	0.39	0.03			
*Bifidobacterium animalis*	NorCA	0.50	0.005			

**FIGURE 4:**
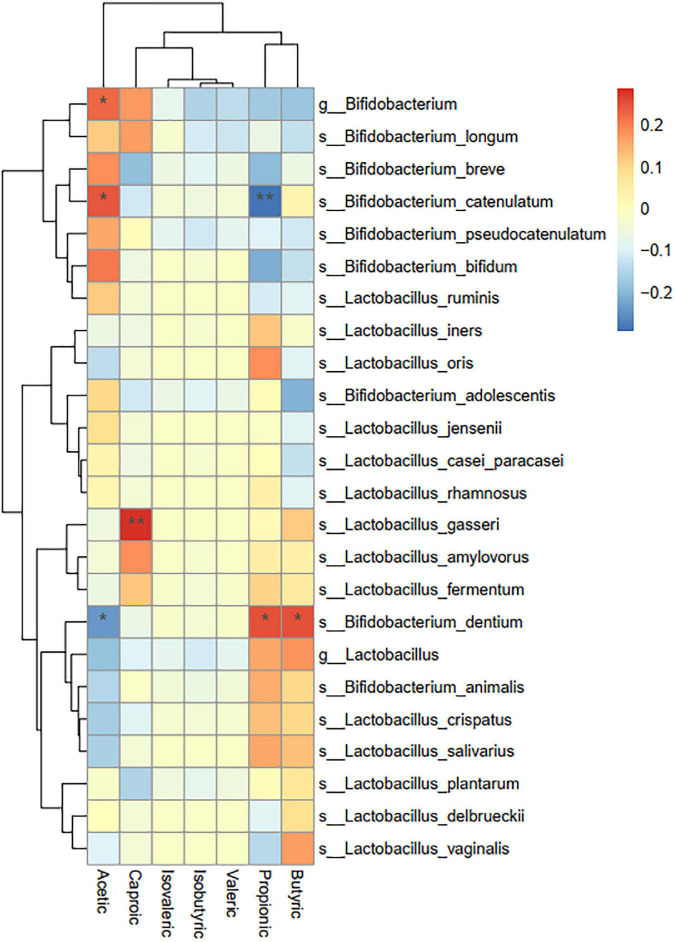
Correlation analysis between probiotics and short-chain fatty acids. Asterisks represent that the corresponding probiotics have a significant correlation with short-chain fatty acids. Red means positive correlation, and blue means negative correlation.

4. A correlation analysis of probiotic species and metabolic indexes of short-chain fatty acids showed that the statistically significant metabolites were mainly acetic acid, butyric acid, propionic acid, and caproic acid, which were significantly correlated with some *Bifidobacteria* (*P* < 0.05) (see Figure 4 and Table 3).

**TABLE 3:** Correlation analysis between probiotics and short-chain fatty acids.

	Positive correlation short-chain fatty acids	Correlation coefficient r	*P*-values	Negative correlation short-chain fatty acids	Correlation coefficient r	*P*- values
*Bifidobacteria*	Acetic acid	0.231	0.03			
*Bifidobacterium* chain	Acetic acid	0.241	0.02			
				Propionic acid	–0.29	0.007
*Bifidobacterium* dentin	Butyric acid	0.24	0.02			
	Propionic acid	0.25	0.02			
*Lactobacillus*				Acetic acid	–0.24	0.02
	Caproic acid	0.24	0.007			

**FIGURE 5:**
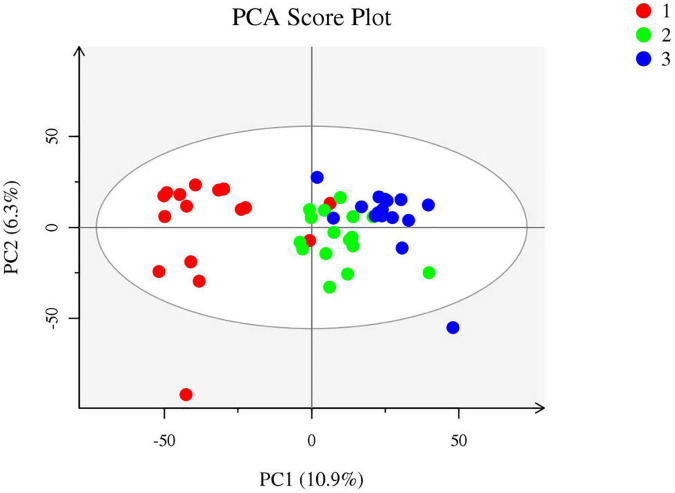
Metabolites with the significant difference in different phototherapy times (1, 2, and 3 represent before phototherapy, 24 h after phototherapy, and 48 h after phototherapy, respectively).

**FIGURE 6:**
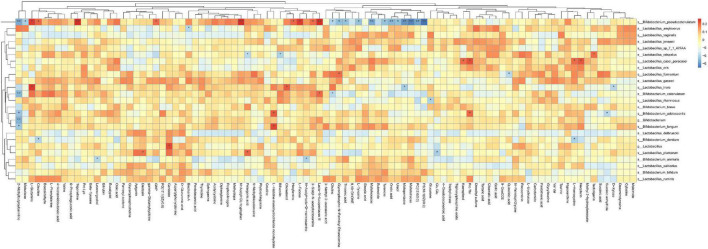
Correlation analysis between changed metabolites and probiotic changes. Asterisks represent that the corresponding probiotics have a significant correlation with short-chain fatty acids. Red means positive correlation, and blue means negative correlation.

5. After phototherapy, 17 metabolites in neonatal intestinal tracts changed significantly, as shown in Figure 5. The correlation analysis of the probiotic species and metabolites also had significant statistical significance, as shown in Figure 6. Table 4 shows the correlation analysis of metabolites with significant changes in probiotics.

**TABLE 4:** Correlation analysis between significantly changed probiotics and metabolites.

Name	DCA	*P*-value	r
*Bifidobacterium longum*	*L*-histidine monohydrochloride monohydrate	0.01	0.23
	2-methylbutyroylcarnitine	0.02	–0.22
*Lactobacillus rhamnosus*	Glucurone	0.03	–0.20
*Bifidobacterium animalis*	*N*-acetyl-beta-*D*-mannosamine	0.03	–0.20
	Levonorgestrel	0.04	–0.19
*Lactobacillus fermentum*	Quinolinic acid	0.04	–0.19
	Glycerophospho-*N*-palmitoyl ethanolamine	0.04	0.19

## Discussion

There are more than 1,000 kinds of bacteria in the human intestinal tract, and the microbial density increases from the proximal to the distal intestinal tract, with a total of about 10^13^–10^14^ microorganisms (6, 7). There are a lot of beneficial bacteria in intestinal microbiota. Probiotics mainly include *Lactobacillus* and *Bifidobacterium*, and their probiotic effects are mainly realised by directly or indirectly adjusting the composition of host intestinal microbes and activating the activity of host endogenous microbial groups or immune systems (8). Probiotics have been widely used in clinical practice. Some scholars believe that probiotics’ participation in the treatment of jaundice is mainly related to regulating intestinal microbiota, lowering intestinal PH value, reducing the quantity and activity of β-glucuronidase, inhibiting the intestinal-hepatic circulation of bilirubin, maintaining intestinal peristalsis, promoting bilirubin excretion, and improving feeding intolerance (9). However, it has not been reported whether the phototherapy process can cause changes in probiotics. Our research found that the abundance of three kinds of probiotics that can be used for newborns decreased significantly after phototherapy, namely *B. breve*, *B. animalis*, and *L. fermentum*. *Bifidobacterium breve* not only had the highest basic abundance but also had the most obvious decline after phototherapy. The decrease in these obvious probiotic species may lead to side effects and even affect children’s long-term flora establishment and metabolism. The influence of these probiotics on the body may play a role through some metabolites.

Many small-molecule metabolites are produced by the co-metabolism of intestinal microbiota and the host, and bile acid is one of them. Intestinal microbiota regulate the synthesis and metabolism of bile acids, which influence the quantity and structure of intestinal microbiota and improve the immune function of a host by regulating the internal inflammatory reaction of the host, and they form a close relationship of mutual influence and regulation (10). Bile acid is an important component of cholesterol metabolism, lipid digestion, and other regulatory pathways of the human body. Dietary and intestinal microbiota interact with bile acid pools and influence the hydrophobicity, toxicity, and regulation of bile acid through biotransformation reactions. Disorders of bile acid pools caused by diseases or temporary antibiotics may lead to various disease states (11, 12). Research by Zampa et al. (13) and other authors has confirmed that when *Bifidobacterium* and *Lactobacillus* are supplemented in the participants’ diet, the bile acid content in the participants’ faeces decreases significantly. Our study found that in addition to the significant changes in probiotics, the content of deoxycholic acid increased after 48 h of phototherapy, which was significantly correlated with *B. breve*. Therefore, after the infants received phototherapy for jaundice, the level of *Bifidobacterium* and *Lactobacillus* in intestinal probiotics decreased, especially *Bifidobacterium brevis*, which weakened their inhibitory effect on the growth of *Clostridium* in the intestinal tract. The increase of *Clostridium* strengthened its dehydroxylation, so the generation of secondary bile acids increased, which led to an increase in intestinal-liver circulation and reduced the effect of phototherapy itself, further providing the theoretical basis for the clinical addition of suitable probiotics.

In the analysis of probiotics and short-chain fatty acid metabolites, it was found that many short-chain fatty acid metabolites decreased as the concentration of probiotics decreased (14). Short-chain fatty acids play an important role in maintaining the normal function of the large intestine and the morphology and function of colon epithelial cells (15). Short-chain fatty acids can directly stimulate the gastrointestinal tract (16), stimulate the secretion of motilin, promote gastrointestinal peristalsis, and accelerate gastric emptying (17). At the same time, an acidic environment can increase osmotic pressure in the intestinal cavity, increase water secretion, reduce the amount of mucus in the faeces, and facilitate defecation (18, 19), thus promoting the excretion of bilirubin with the faeces, reducing bilirubin levels *in vivo*, and relieving jaundice to some extent. Our research shows that phototherapy leads to a decrease in probiotics, and the short-chain fatty acids positively related to it will also decrease, which may lead to the positive effect of short-chain fatty acids affected by phototherapy, resulting in clinical side effects.

At the same time, through the analysis of metabolites, we found that the metabolites also changed significantly after phototherapy, and there were many metabolites that increased or decreased significantly after phototherapy. Many metabolites that were closely related to the significant changes in probiotics after phototherapy are involved in the metabolism of nutrients in the body. For example, *L*-histidine hydrochloride is a chemical substance that is mainly used as a nutritional supplement and belongs to the quasi-essential amino acids (essential amino acids for infants and young children), and it is synthesised slowly in the human body. Its deficiency can lead to symptoms such as growth retardation and eczema, and this metabolite is positively correlated with *Bifidobacterium longum*. A metabolite negatively related to *B. animalis* is *N*-acetyl-*D*-mannosamine (ManNAc). ManNAc is an essential precursor of *N*-acetylneuraminic acid, and the specific monomer of bacterial capsular polysialic acid is involved in the occurrence of neurological diseases. Significantly negatively related to *L. fermentum*, quinolinic acid (20) is an endogenous NMDA receptor agonist that is synthesised from *L*-tryptophan through the kynurenine pathway, so it has the potential to regulate the injury and dysfunction of *N*-methyl-*D*-aspartate neurons. The probiotic changes caused by phototherapy may indirectly affect the metabolism of newborns through the above metabolites.

The metabolism of bilirubin in newborns is closely related to the intestinal microenvironment, and an intestinal microbiota disorder can aggravate the progress of jaundice and even affect the therapeutic effect of phototherapy. As a routine treatment for neonatal jaundice, phototherapy has some side effects, such as fever, diarrhoea, rash, and bronchus. Our previous research found that phototherapy causes changes in the neonatal flora of jaundice, which may be one of the reasons for the side effects of phototherapy. At present, there is no uniform probiotic selection standard for newborns with jaundice who receive phototherapy. Our research focuses on the changes in the intestinal probiotic species and the metabolism of newborns receiving phototherapy. It was found that phototherapy can significantly reduce the content of important probiotics in the body and also affect the metabolism of bile acids, which provides a theoretical basis for the clinical, targeted selection of appropriate probiotics.

## Data availability statement

The original contributions presented in this study are included in the article/supplementary material, further inquiries can be directed to the corresponding author.

## Ethics statement

The studies involving human participants were reviewed and approved by Ethics Committee of Shanghai Sixth People’s Hospital East Campus (Approval No: 2020-071). All family members signed an informed consent form. Written informed consent to participate in this study was provided by the participants’ legal guardian/next of kin.

## Author contributions

SF and JPZ: conception and design of the research. JHZ, KZ, and FZ: acquisition of data. LZ, LL, and ZW: analysis and interpretation of the data. AL: statistical analysis. JPZ: obtained financing. SF and YM: writing of the manuscript. XF: critical revision of the manuscript for intellectual content. All authors contributed to the article and approved the submitted version.
